# Telehealth Services for Children With Autism Spectrum Disorders in Rural Areas of the Kingdom of Saudi Arabia: Overview and Recommendations

**DOI:** 10.2196/11402

**Published:** 2018-11-15

**Authors:** Shahad Alkhalifah, Hesham Aldhalaan

**Affiliations:** 1 Centre for Autism Research King Faisal Specialist Hospital & Research Centre Riyadh Saudi Arabia

**Keywords:** autism spectrum disorders, intervention, Saudi Arabia, telehealth

## Abstract

Autism spectrum disorders (ASD) are the most-prevalent neurodevelopmental disorders. However, each child diagnosed with ASD presents with a unique range of behavioral and communication problems and issues with social skills. Many studies have highlighted the importance of early interventions for children with ASD to improve their skills and provide their families with the necessary support. However, in the Kingdom of Saudi Arabia (KSA), the earliest that a child with ASD in the major cities receives an intervention is at the age of 4 years, owing to limited services and a lack of awareness of the importance and benefits of early interventions. Families who live in rural areas of KSA arguably have a greater need for these services, as they have to travel to cities such as Riyadh for help. The use of telehealth services may be effective for ASD intervention among children living in rural areas, since such services use technology to provide consultations, interventions, diagnosis, training, and education. Research indicates that telehealth services are as valuable as traditional face-to-face treatment, allow families to obtain support from their homes, and help them improve their quality of life. This review will discuss the application of telehealth services to support families in rural areas of KSA who are dealing with issues of ASD, considering the cultural and religious contexts. In addition, it will examine ways in which technology can be employed to suit KSA’s culture and needs.

## Introduction

Autism Spectrum Disorder (ASD) is the most-widespread developmental disability: Its global prevalence has dramatically increased from a rate of 1 per 10,000 people in the 1980s to 1 per 68 people in 2016 [1]. However, in the Kingdom of Saudi Arabia (KSA), there are no data about the number of cases of ASD, although it is estimated to be over 167,000 in a population of over 28 million people [2]. In addition, the services available for children with ASD in the cities do not meet the demand; as a result, some families are forced to travel to neighboring countries such as Jordan or overseas to countries such as the United States for these services [2]. Research worldwide aims to continually develop ways to improve interventions in order to ensure better lives for children with ASD and their families [3]; however, KSA is significantly lagging in relevant developments [4], because the methods developed in the West are not usually culturally suitable for KSA, particularly with regard to prescribed gender roles [1]. However, one method that might be effective is telehealth services. From this view point, we will review evidence from countries outside KSA that prove the effectiveness of telehealth in providing services to children with ASD. In addition, we will discuss the potential applications of this method in the Saudi context, and the challenges it may pose, in particular, due to cultural specificities. Further, we will provide recommendations to overcome these barriers. We performed a narrative literature review, as this is a useful method to develop recommendations for clinical practice [5]. Relevant literature was identified through an electronic keyword search of four databases (PubMed, Web of Science, Cumulative Index to Nursing & Allied Health Literature, and Scopus) using the search terms “telehealth,” “family-centered approach” or “technology,” in combination with the term “autism” (using the AND Boolean term), and dated up to April 2017. Broad search terms were used for their relevance to the research question and to reduce selection bias. However, the search was confined to peer-reviewed papers, as the literature review was not systematic; additionally, unpublished and grey literature was not considered appropriate for clinical practice, due to its low standard of evidence.

## Telehealth Services for Children with Autism Spectrum Disorders

The most evidence-based intervention for children with autism is early behavior intervention [6], which has several advantages including its ability to be delivered by anyone with training, not only practitioners with advance degrees (such as clinical psychologists or therapists). Therefore, in early behavior interventions, even caregivers are practitioners, provided they have received training. In addition, the use of a family-centered approach to educate and support caregivers of children with ASD has been found to improve outcomes for children [7]. In this approach, the caregivers’ needs are taken into account, and caregivers are trained to interact with children with ASD, without therapists assuming the knowledge of caregivers [8]. Russa and colleagues [9] concluded that ASD interventions are interdependent on caregivers and that active engagement of caregivers is key to positive outcomes. However, delivery of early behavior interventions and family-centered services face-to-face poses many barriers such as the scarcity of trained therapists, limited resources and services, low socioeconomic status of many families, lengthy waiting lists, and practical issues arising from the fact that these limited services are only available in major cities [10]. Therefore, it is important to determine ways to help caregivers adapt new methods to deliver interventions for ASD [10]. Due to the significant increase in the use of the computer and Internet in everyday life, telehealth services, which use technology to provide services from a distance to families with a child with ASD, could be an alternative and effective method of providing support [10]. This method has many advantages. First, the family can interact with an instructor directly via video, which potentially provides access to a greater range of expert therapists. Second, by providing caregivers with an opportunity to play an active role in the child’s development, telehealth technology can empower caregivers and accelerate the diagnostic process [10,11].

Telehealth consists of a range of computerized software applications such as video conferencing, digital versatile discs, three-dimensional interactive programs, mobile phone apps, and telephone- and web-based tutorials [12]. Studies of telehealth-based parent training showed that the caregivers found the training programs convenient, practical, appropriate, and helpful for increasing their knowledge about evidence-based intervention methods [13-15]. In addition, studies reported positive changes in children’s outcomes [3,10,11,16]. For instance, a study of North American families with children aged < 48 months who had ASD revealed that teaching parents the Early Start Denver Model intervention through videoconferencing and a web tutorial increased the rates of vocalization and joint attention initiation in children [3]. Similarly, a study of children aged 24-72 months with ASD in North America found that teaching parents an imitation intervention through remote coaching and self-directed online study increased spontaneous imitation in the children [10]. However, most studies included a small to very-small sample size; the only study with a reasonable sample size [13] did not measure children’s outcomes and implemented the training with caregivers and professionals who worked with children with ASD (eg, teacher assistants). Another limitation is that the studies mostly included college-educated participants, although the principles of early behavior intervention state that it can be implemented even if one does not have an advance degree [13]. This limitation will be further discussed in the context of KSA.

## Barriers and Challenges in Adopting Saudi Telehealth Services for Children with Autism Spectrum Disorders

In KSA, families of children with ASD, particularly those living in rural areas, encounter many difficulties when seeking support [4]. As mentioned earlier, the number of children with ASD is increasing, but services are only available in major cities. In addition, due to the limited resources, waiting times for appointments or family-centered approach sessions can take 8-12 weeks [17]. Moreover, several aspects, including the costs of travel and the service itself as well as the need to manage receipt of support for their child while maintaining jobs and fulfilling other responsibilities, lead to a significant strain on families living in rural areas of KSA [2,17,18]. For such families, telehealth could address many such issues and provide additional support to existing services. Telehealth services could be used as a tool for teaching strategies to improve the outcomes of children with ASD and their families [17,19]. However, the cultural context, beliefs, educational levels, and socioeconomic status of the KSA population need to be considered when selecting appropriate methods of intervention [18]. Furthermore, because telehealth services employ technology in a new way, research is required to identify a reliable, evidence-based telehealth framework and intervention program in order to deliver services from a distance in KSA.

The first issue is that in KSA culture, women assume the role of a homemaker and carer, whereas men provide for and protect the family, leaving the mothers with almost complete responsibility of caring for the children [20]. In addition, KSA culture dictates that men and women should always be separated (eg, in education, banking, and health), women should cover their faces in front of men, and some women are not allowed to have their picture taken [20]. These rules make communication between genders a sensitive issue [21]. In fact, face-to-face contact, including online communication, between men and women is prohibited by culture [21]. Therefore, in some cases, interventions or interviews with mothers of children with ASD are not allowed to be conducted by men without the presence of the mother’s relatives [22]. This reduces opportunities for teleconference or video-conference for face-to-face sessions between the therapist and the child’s mother, which could otherwise help the therapist provide recommendations, answer questions, and design an intervention strategy while observing the child through a camera in the child’s home [23].

The second obstacle to the use of technology to provide assistance to families affected by ASD is the prerequisite that the family have a computer or mobile phone that can be used to access the internet, a high-speed internet connection, and information technology literacy [19]. Although 40% of KSA families living in rural areas are living below the breadline [24], by 2012, 95% of Saudi individuals had a mobile phone and mobile internet penetration was 70% [25]. As such, it is unlikely that parents of children with ASD do not have access to the internet, or at least a mobile phone, in some form.

The use of telehealth programs to assist families with a child with ASD requires training [19]. Thus far, studies that examined the effectiveness of telehealth in the context of ASD mostly recruited educated participants. However, KSA has overall poor education levels [18]. In rural areas, many mothers of children with ASD do not have a degree and have often not attended high school [18]; some of them are illiterate, which makes the use of technology extremely difficult [2,18,22]. In addition, the Saudi government does not provide sufficient education on the use of technology. Therefore, institutions such as public schools in rural areas are often unaware of the latest advances in technology [24].

The third problem with implementing a telehealth service in KSA is the need for Saudi mothers, as the principal carers, to talk to female, rather than male, therapists [26]. Although the health system allows a mix of genders in KSA, this is a male-dominated field with a low number of Saudi women [20,27]. Thus, the majority of female professionals in the KSA healthcare system are foreign workers, which often results in problems with the delivery of information because a translator, who is usually a non-expert, is needed [20,27]. In addition, many online applications are not in Arabic, and therefore, a team is required to translate the applications into Arabic. Furthermore, Saudi expertise in technology is limited [27].

Finally, the use of telehealth services requires caregivers to act as the child’s ASD therapist, managing new strategies for care and using different technological methods of intervention from professionals [10]. However, Saudi families, especially mothers, historically have profound trust in healthcare professionals, which might deter them from assuming such an influential role in their child’s intervention without a healthcare team [28].

In summary, introduction of technology to support families living with ASD in rural areas has many advantages, including flexibility. However, KSA is a traditional country where Islamic teachings and Arabic cultural values are strictly followed; therefore, such telehealth services need to be selected and implemented carefully. In addition, cultural factors such as the need to hire both male and female Arabic-speaking educators, gender roles, and educational levels need to be considered.

## Recommendations for Best Practice

Since telehealth services have recently been introduced in KSA, many of the abovementioned barriers could prove detrimental to the success of this service. However, there is an evident need to help families with children with ASD who are living in rural areas [4]. The world revolves around technology, which enhances social interaction; facilitates knowledge sharing among health professionals, individuals with ASD, and the whole community; and will lead to positive change in the health sector (eg, through treatment plans) [28]. For example, the use of mobile internet technology such as WhatsApp has shown promising results in reducing stress through interventions in Saudi parents of children with ASD [29].

Considering the recent evidence, the government and health minister need to implement these telehealth services to assist families affected by ASD, through the use of technology, engaging both genders in the use of computers and up-to-date software. In addition, an increase in socialization between genders and the fact that this change might benefit children with ASD could positively affect the development of children and families by improving access to quality medical and behavioral services in the healthcare system [4]. However, occasionally, the distribution of physical training materials (eg, printed manuals) may be required to educate people in rural areas without internet access. Nonetheless, not all rural regions of KSA lack access to the internet, and as highlighted above, mobile phone ownership is now widespread, with extensive mobile internet penetration [25]; it would therefore be valuable to consider the use of telehealth services for such regions.

Many Saudi caregivers reported that their children were diagnosed with autism at the age of 7 years, because the hospitals in their area were not trained sufficiently to diagnose autism [22]. The caregivers had to refer the children to big cities for a diagnosis, which took months because of the limited number of professionals with the ability to diagnose the disorder. Evidence indicates that early intervention improves children’s outcomes, and a delay in diagnosis can lead to a delay in early intervention services [28,30]. In this regard, the use of the Naturalistic Observation Diagnostic Assessment (NODA) SmartCapture, a mobile phone app for caregivers to record video evidence of a child’s behavior in their natural environment (eg, the home), could help diagnosticians confirm the diagnostic assessment of ASD [11]. Using NODA, caregivers place the child in specific social scenarios to ensure that the recorded videos provide professionals with the necessary elements for a diagnosis [31]. The scenarios were designed with reference to the Diagnostic and Statistical Manual of Mental Disorders, 5th edition, criteria [32]. Preliminary research showed 91% agreement between diagnoses made using NODA and those made face-to-face previously by another professional [11].

The NODA method presents specific advantages for rural populations. First, families do not need to purchase relatively expensive equipment and pay for travel expenses, which makes the method cost effective and consequently, much more attractive than face-to-face visits [10,11]. In addition, families of children with ASD, who may dislike changes to their routine and social context, do not need to travel to large, unfamiliar cities, which evidently reduces stress [10]. Moreover, online assessment through videos would be more ecological, as the child would be in his or her natural environment. Although online assessments may lack reliability, Smith et al. [33] reported similar results between online and face-to-face clinician assessments of ASD in a prospective study of 50 families (88.2% agreement). Furthermore, 95% caregivers reported that NODA SmartCapture was easy to use and they were able to record videos in their homes successfully [34]. Therefore, NODA SmartCapture could improve the efficiency of the diagnostic process for ASD in KSA.

The uneven internet access in KSA could hamper the effectiveness of NODA in rural areas. Professionals should therefore consider the use of telephone services for diagnoses, as a standby option [16], as 95% of the population owns mobile phones [25]. Professionals could administer the Autism Diagnostic Interview-Revised (ADI-R) via the telephone [35]. The ADI-R is the gold standard test for the diagnosis of ASD. Research in diagnoses made by ADI-R showed that there is no significant difference between face-to-face interviews and those performed over the phone [35]. Once an ASD diagnosis has been established, we recommend that the family visit a major city for further assessment (eg, intellectual assessment), as there is limited evidence that these assessments can be performed via telehealth for ASD in young adults [36]. Although it is inconvenient that people receiving a diagnosis remotely need to travel to a major city for an intellectual assessment, this method will minimize the number and cost of trips and can therefore reduce the average age at which people in rural areas are diagnosed, thereby facilitating early interventions. Once a full assessment is conducted, the aim is to provide further support for interventions through telehealth (and occasionally printed materials), which will further reduce the number for trips needed. Moreover, remote consultations could minimize the burden on hospitals in major cities by identifying people whose behaviors are not symptomatic of ASD and who therefore do not need in-person consultation.

Second, web-based learning could be of immense help to the families of children with ASD after diagnosis, as it could potentially include video tutorials that highlight specific ASD behaviors and advise caregivers on the correct use of intervention strategies [37]. In KSA, the use of web-based learning is theoretically valuable and flexible, as it can be adapted to suit the cultural context of families of children with ASD [37]. In addition, although male therapists in KSA cannot train female caregivers face-to-face about behaviors of children with ASD, it would be acceptable for women to watch learning videos made by male therapists, as this does not represent direct contact. This is an additional advantage of telehealth in the Saudi context: The use of learning videos will allow therapists to train a wider population outside the major cities and reduce reliance on female trainers, which is necessary for delivering face-to-face training to female caregivers. Therefore, for Saudi female caregivers who cannot read or understand medical terms, such videos would be immensely useful. The website could also have a forum either using email or a method similar to email, so that caregivers can send and receive voice or text messages to their therapist, who could then provide instantaneous advice and support [3]. Since the Saudi culture is strict about communication between genders, the forums should be moderated by female staff through whom communication between female caregivers and male therapists could occur.

Another method would be to develop a website that provides caregivers and the general public with resources such as information on autism symptoms, educational brochures, early interventions programs, strategies for dealing with issues at home, community activities, and local events [3]. In addition, it should allow caregivers to post anonymous conversational topics, tips, and progress updates about their child or any other information they may want to share [3]. In KSA, such a method could help resolve the separation issue between the genders, thereby helping them support each other, increasing awareness of other local families of children with ASD, and keeping them up-to-date on new interventions for ASD. This method would also help raise public awareness of ASD symptoms [22] and increase the probability of caregivers seeking treatment early, even in rural areas. As mentioned earlier, online training for families of children with ASD were as effective as face-to-face workshops [38].

Telehealth services could also benefit health professionals. Alqahtani [22] stated that many ASD therapists in KSA are not trained for the latest intervention strategies; as such, web-based learning could help update them on the latest research and intervention techniques [16]. For example, a preliminary study by Vismara et al. [38], including 10 therapists, showed that training therapists to use the Early Start Denver Model for ASD via telehealth was particularly effective, and Hamad et al. [13] found that a large sample of training therapists could significantly improve knowledge about early behavior intervention after e-learning.

Finally, a few different interventions have been tested through telehealth in recent years [39]. In most studies, the outcomes were positive [37,39], suggesting that telehealth could be applied to most interventions in practice. Providing caregivers with online coaching is conducive to achieving optimal outcomes. For instance, Ingersoll and Berger [40] found that parents of young children (aged 27-73 months) were more likely to engage in a social communication intervention (ImPACT Online) if, in addition to completing online lessons, they participated in 30-minute videoconferences with therapists twice per week, as compared to parents who only completed the online lessons. Notably, engagement in the group receiving only online lessons was high, and the lessons included a range of media such as slideshows, videos, quizzes, and reflective questions. Similarly, Pickard et al. [41] observed that in comparison to parents who completed a self-directed online study, parents who received additional online coaching via Skype were more likely to report improvements in communication in children aged 19-73 months. Lindgren et al. [42] reported that delivery of functional communication training through telehealth in the caregiver’s home is as effective as that in a regional clinic and in-home therapy with respect to reducing problematic behaviors in children aged 21-48 months. However, home-based telehealth is the most affordable of these approaches. Collectively, these results suggest that online web-based lessons that use a range of media represent an effective and comparatively cost-effective method to improve outcomes in children with ASD and that their efficacy might be enhanced by online coaching. However, further research is needed to directly compare telehealth interventions in order to recommend the most-effective option; this is even more important in light of publication bias, through which results less favorable than those of telehealth might not be published [39,43].

## Conclusion

Although autism is becoming increasingly recognized as a disorder in KSA, there are limited services for autism in rural areas. Increased public awareness about the disorder is needed to meet the demand for adequate services, support, and research that are suitable to the Saudi culture. Although services and support can be found in major cities, this is not the case throughout the country. Alternative methods for assisting families dealing with ASD can be potentially identified through technology. However, the use of technology involves many obstacles due to KSA’s culture and religion. Adapting the technology accordingly could make a significant difference to the families with children with ASD. Moreover, telehealth services could allow healthcare professionals in KSA to interact with caregivers in rural areas, providing consultations, behavioral interventions, and other support. Furthermore, such methods may lead to more acceptance use of technology in society, in general. However, for this progressive, yet effective, method of support to gain momentum in KSA, research on its implementation is urgently required.

## Abbreviations

ADI-R: Autism Diagnostic Interview-Revised
ASD: Autism Spectrum Disorder
KSA: Kingdom of Saudi Arabia
NODA: Naturalistic Observation Diagnostic Assessment
